# A Review on Map-Merging Methods for Typical Map Types in Multiple-Ground-Robot SLAM Solutions

**DOI:** 10.3390/s20236988

**Published:** 2020-12-07

**Authors:** Shuien Yu, Chunyun Fu, Amirali K. Gostar, Minghui Hu

**Affiliations:** 1 State Key Laboratory of Mechanical Transmissions, School of Automotive Engineering, Chongqing University, Chongqing 400044, China; shuien_yu@cqu.edu.cn (S.Y.); hu_ming@cqu.edu.cn (M.H.); 2 School of Engineering, RMIT University, Melbourne, VIC 3001, Australia; amirali.khodadadian@rmit.edu.au

**Keywords:** multi-robot SLAM, map merging, occupancy grid map, feature-based map, topological map

## Abstract

When multiple robots are involved in the process of simultaneous localization and mapping (SLAM), a global map should be constructed by merging the local maps built by individual robots, so as to provide a better representation of the environment. Hence, the map-merging methods play a crucial rule in multi-robot systems and determine the performance of multi-robot SLAM. This paper looks into the key problem of map merging for multiple-ground-robot SLAM and reviews the typical map-merging methods for several important types of maps in SLAM applications: occupancy grid maps, feature-based maps, and topological maps. These map-merging approaches are classified based on their working mechanism or the type of features they deal with. The concepts and characteristics of these map-merging methods are elaborated in this review. The contents summarized in this paper provide insights and guidance for future multiple-ground-robot SLAM solutions.

## 1. Introduction

Autonomous driving technologies have been developing rapidly in recent decades. Environment perception, path planning, and motion control are considered the three core technologies that enable autonomous driving [1,2]. In an autonomous vehicle, environmental perception is achieved by means of onboard sensors and computer units, aiming to replace the human’s perception system. The primary problems to be solved for environment perception are as follows: (1) where am I (localization) and (2) what is around me (mapping) [3]? These two problems are indeed intertwined and must be solved simultaneously, since the accuracy of vehicle localization directly affects the accuracy of mapping and vice versa. This requirement has driven forward the research on simultaneous localization and mapping (SLAM), which has attracted a lot of attention from both the robotic and automotive communities [4,5].

The focuses of current SLAM research are being shifted from indoor environments to outdoor environments, and also from small-scale simple static scenes to large-scale complex dynamic scenes. Thus, it is becoming increasingly difficult to complete the SLAM task using only a single robot. Driven by this challenge, more attention is now being paid to multi-robot SLAM (or multi-vehicle SLAM) due to its high-efficiency [6,7,8], which involves more than one robot/vehicle working collaboratively in a certain environment. Multi-robot SLAM can significantly improve mapping efficiency, as the maps produced by different robots are merged to form a greater map, which avoids repeated exploration of the same place. However, the existing multi-robot SLAM solutions in the literature are mostly based on the achievements of single-robot SLAM approaches. The type and quality of the maps generated by individual robots vary greatly due to the differences of sensors on individual robots, and as a result, direct fusion of these individual maps becomes very challenging [9].

**Figure 1:**
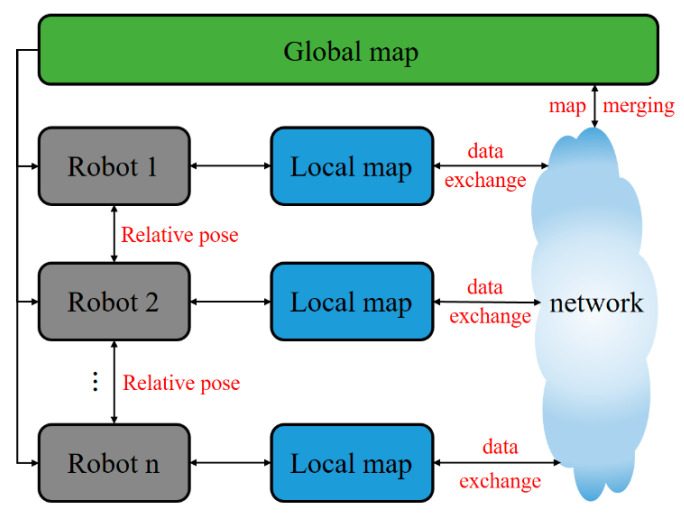
Three major tasks for multi-robot simultaneous localization and mapping (SLAM).

**Figure 2:**
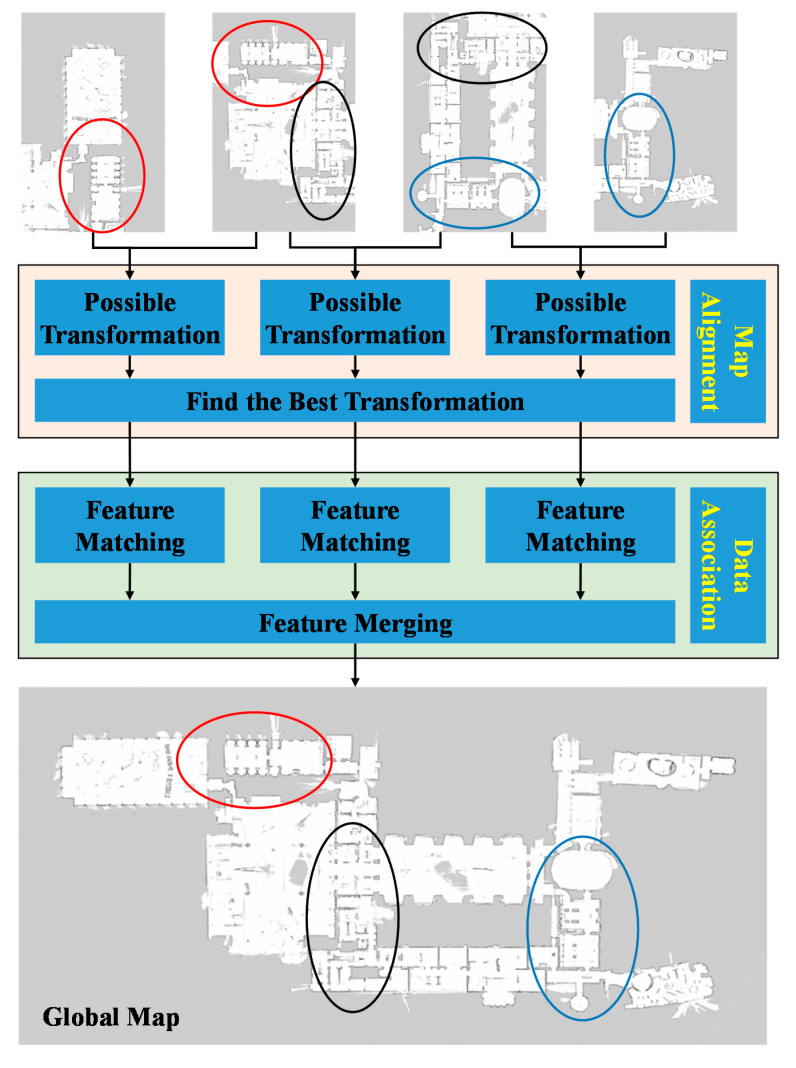
Example map-merging process. Four local maps are merged to construct a global map of the environment. The common area of the first and second local maps are represented by red ellipses. The common area of the second and third local maps are represented by black ellipses. The common area of the third and fourth local maps are represented by blue ellipses.

When a group of autonomous robots enter a large-scale environment (such as an unknown hazard site), the multi-robot system needs to collaboratively explore the unknown environment autonomously. The following three major tasks need to be accomplished for multi-robot SLAM: determination of relative poses, data exchange (communication), and map merging (or map fusion), as shown in Figure 1. Determination of relative poses is referred to as the acquisition of robot poses with respect to a specific robot, before or during the process of collaborative exploration [10]. Relative poses of robots are crucial to the collaborative exploration of multiple robots. With the knowledge of relative poses and local maps created by individual robots, repeated exploration of the same position can be avoided, and the efficiency of mapping can be significantly improved. As for data exchange, short-range communication technologies such as Bluetooth [11], RFID, and WIFI [12,13] can be used for communication between robots. Two crucial steps involved in map merging are map alignment and data association [14]. Map alignment is the process of finding appropriate spatial coordinate transformations between local maps. This process should be distinguished from the determination of relative poses. The former one is to calculate the coordinate transformation between partial maps, while the latter one is to determine the pose relationship between different robots. Many of the existing map alignment algorithms are devised based on some assumptions, such as similar map formats, same map scale, and large map overlapping ratio [15,16,17]. The purpose of data association is to match and merge the features between partial maps, which is a crucial step for fusing the maps established by multiple robots. The complete map-merging process is shown in Figure 2.

In recent years, several reviews on multi-robot SLAM have been presented in the literature, such as [2,9,18,19,20]. The emphases of these works differ from each other: Saeedi et al. [9] first briefly introduced the existing single-robot SLAM algorithms and then explained the issues with multi-robot systems. The map-merging problem is not explained in detail in this work. Rone and Ben-Tzvi [2] summarized three key problems existing in multi-robot systems: mapping, localization, and motion planning, but this work lacks a systematic introduction to map merging. Amigoni et al. [19] demonstrated the importance of communication for autonomous mobile robots and studied existing solutions under different communication conditions. However, the map-merging problem, which is significantly affected by communication, has not been investigated in detail in this work. Andersone [20] summarized the latest research in homogeneous and heterogeneous map merging and pointed out six important factors that influence map merging. The state-of-the-art multi-robot SLAM solutions are summarized in the above works from different perspectives. However, one important aspect is missing in these review works: the details of map-merging methods for different map types. To supplement the above review works on multi-robot SLAM and provide the readers with a more comprehensive understanding in map fusion, in this paper, the map-merging methods for three typical map types are reviewed in detail.

In the existing literature, the surrounding environment is commonly represented by three major types of maps [2]: occupancy grid maps, feature-based maps, and topological maps. These three maps are the most typical forms of map representation in the relevant literature, and there have been a lot of relevant research results. Therefore, this paper mainly reviews the map-merging methods for these three types of maps. Note that the research on these maps started at different times in history, which makes the popularity and number of available works in the literature different from each other. Hence, the contents summarized for these three types of maps are not equal in this review. Besides, other maps, such as appearance-based maps, random probability maps, and semantic maps, are not widely used in SLAM applications and are not discussed in this paper.

The remainder of the paper is organized as follows. Section 2 introduces the merging algorithms for occupancy grid maps, Section 3 reviews the merging methods for feature-based maps, Section 4 investigates the merging approaches for topological maps, and Section 5 concludes the paper.

## 2. Occupancy Grid Map Merging

**Figure 3:**
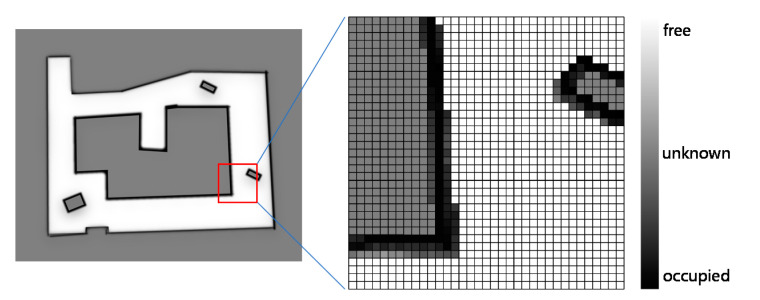
An occupancy grid map example.

An occupancy grid map is a representation of the environment in which the environment is divided into a series of grids. In an occupancy grid map, each grid is given a value (known as the grid occupancy) to reflect the probability that the grid is occupied. At any time, each grid is in one of the following three states: occupied, free, or unknown [10,21]. These three states normally correspond to three intervals of probabilities. For example, a probability value between 0.9 and 1 represents “occupied”, and that between 0 and 0.1 denotes “free”. All other probability values reflect an unknown state. As seen in Figure 3, the gray color represents the unknown area, the black color represents the occupied area (i.e., obstacles exist in the grids), and the white color represents the free area in which the robots can move freely.

Compared with other types of maps used in SLAM, the occupancy grid map presents the following advantages: (1) the occupancy grid map is suitable for building unstructured environment [10], (2) the principle of occupancy grid map is straightforward [22,23], (3) the mapping speed is fast as no environmental features are extracted [24], and (4) the resolution of an occupancy grid map can be easily adjusted by changing the size of grids. On the other hand, one shortcoming of the occupancy grid map is that if a high map resolution is chosen or the environment becomes large, then big storage space and high computation power are necessary [3,22].

Significant differences are present in the existing merging methods for occupancy grid maps. In this section, these merging methods will be classified based on their distinct working mechanisms and introduced in different subsections. The methods in Section 2.1 only employ probabilistic methods for map-merging purposes. The concept of optimization function is utilized in the approaches in Section 2.2 to merge occupancy grid maps. The works in Section 2.3 first extract features from occupancy grid maps and then conduct feature matching to merge maps; the Hough transform-based method works in an obviously different manner from the others, which is separately introduced in Section 2.4.

### 2.1. Probability Method

Since occupancy grid maps use probabilities to indicate the occupancy state of each grid cell, it is intuitive to employ probability methods to merge multiple occupancy grid maps [25,26]. Assuming that two robots know each other’s initial poses, merging the map with the probability methods is equivalent to estimating the posterior of the robot trajectories and the global map, whose process can be mathematically expressed as follows [27]:

(1) $$\begin{array}{l} p(x_{1:t}^{1},x_{1:t}^{2},m|z_{1:t}^{1},u_{0:t-1}^{1},x_{0}^{1},z_{1:t}^{2},u_{0:t-1}^{2},x_{0}^{2})=\\ p(m|x_{1:t}^{1},z_{1:t}^{1},x_{1:t}^{2},z_{1:t}^{2})p(x_{1:t}^{1}|z_{1:t}^{1},u_{0:t-1}^{1},x_{0}^{1})p(x_{1:t}^{2}|z_{1:t}^{2},u_{0:t-1}^{2},x_{0}^{2}) \end{array}$$

where $x_{1:t}^{1}$ denotes the trajectory of robot 1, $x_{1:t}^{2}$ denotes the trajectory of robot 2, $z_{1:t}^{1}$ denotes the measurement of robot 1, $z_{1:t}^{2}$ denotes the measurement of robot 2, $u_{0:t-1}^{1}$ denotes the control input of robot 1, $u_{0:t-1}^{2}$ denotes the control input of robot 2, $x_{0}^{1}$ denotes the initial pose of robot 1, $x_{0}^{2}$ denotes the initial pose of robot 2, and m denotes the global map. Under the assumption that the trajectories and observations of two robots are independent of each other, the left part of Equation (1) can be further factored to the form of the right part. The first term on the right gives the map density with known robot trajectories and observations, and the remaining two terms are the trajectory densities of robot 1 and robot 2, respectively.

Equation (1) gives a general description for building a global map, when two robots know each other’s initial positions. This equation can be easily extended to a more general case where n≥2 robots are involved. An application is introduced in [28] with known initial robot positions, and the occupancy grid maps contributed by different individual robots are merged using the following equation:

(2) $$P(occ_{x,y})=\frac{odds_{x,y}}{1+odds_{x,y}}$$

with

(3) $$odds_{x,y}=\overset{n}{\underset{i=1}{\Pi }}odds_{x,y}^{i}$$

(4) $$odds_{x,y}^{i}=\frac{P(occ_{x,y}^{i})}{1-P(occ_{x,y}^{i})}$$

where $P(occ_{x,y})$ denotes the probability of grid at position (x,y) being occupied by obstacles, and $P(occ_{x,y}^{i})$ represents the probability of grid at position (x,y) being occupied by robot i in the global coordinate system.

When the initial relative poses of robots are known, the multi-robot map-merging problem is indeed an extension of the single-robot situation [29]. However, in many cases, knowing the initial relative poses is a challenging assumption, which can hardly be satisfied. As a result, many studies have been focused on situations where the initial relative poses are unknown [11,30]. The relative poses can be determined as the robots approach each other. When the robots are within the communication range, the relative poses are measured and the map information (or raw measurement data) is exchanged, after which their individual maps are merged into a global map. This process can be expressed mathematically as follows [27]:

(5) $$\begin{array}{l} p(x_{1:t}^{1},x_{s+1:t}^{2},m|z_{1:t}^{1},u_{0:t-1}^{1},x_{0}^{1},z_{s+1:t}^{2},u_{s:t-1}^{2},\Delta_{s}^{2})=\\ p(m|x_{1:t}^{1},z_{1:t}^{1},x_{s+1:t}^{2},z_{s+1:t}^{2})p(x_{1:t}^{1}|z_{1:t}^{1},u_{0:t-1}^{1},x_{0}^{1})p(x_{s+1:t}^{2}|z_{s+1:t}^{2},u_{s:t-1}^{2},\Delta_{s}^{2}) \end{array}$$

where $\Delta_{s}^{2}$ represents the relative pose of robot 2 with respect to robot 1, measured by robot 1 at time s. Note that s is the moment when two robots encounter for the first time, and other symbols in this equation have the same meaning as in Equation (1).

Although the above works can accomplish the task of map merging for multiple robots with unknown initial poses, they either do not take into account the communication between robots at all, or they simply assume that the communication is in good condition. To ensure the robustness of multi-robot systems, a more practical and challenging situation needs to be considered, where the local maps are merged when the initial poses are not known, and only limited communication support is in place. An exploration strategy for limited communication conditions is proposed in [31] and [32]. The core idea of this strategy is that when the robots are not within the communication range, they construct their local maps on their own; when any two robots get close enough and start communicating, they share their local maps and then one robot locates the other in its own map. Carlone et al. [3,11] looked into the multi-robot SLAM problem under the condition of limited communication. In their approach, any two robots are assumed to communicate only when they are close enough, and only two robots can communicate at any time. A robot first estimates its own position and builds a local map by means of filtering, and then it adjusts its position and local map once other robots’ information becomes available. The feasibility of this method is verified through both simulation studies and experimentation, and this method can be applied to multi-robot systems with typical short-distance communication technologies such as Bluetooth, WIFI, and RFID.

### 2.2. Optimization Method

The concept of optimization is also employed by researchers for solving the map-merging problem. Optimization normally means maximizing or minimizing an objective function under certain constraints. The key step for solving the map-merging problem, using the concept of optimization, is to design an appropriate objective function. Some important objective functions are illustrated in this section.

#### 2.2.1. Objective Function Based on Overlapping

When dealing with occupancy grid maps, the perceived environment is divided into a large number of rectangular units of equal size in the mapping process. Each cell in the occupancy grid map contains an occupied probability to indicate its status. These probabilities are discretized and stored in a matrix, which can be expressed as [21,33]:

(6) m:[0,N]×[0,M]

where N, M are two positive real numbers, and m is a matrix that stores integer numbers.

The rigid transformation (including rotation and translation) between two maps is given by:

(7) $$T_{tx,ty,\theta (x,y)}=\left[\begin{array}{ccc} \cos\theta & -\sin\theta & tx\\ \sin\theta & \cos\theta & ty\\ 0 & 0 & 1 \end{array}\right]\left[\begin{array}{c} x\\ y\\ 1 \end{array}\right]$$

where θ represents the rotation angle between the two maps, $t_{x}$ and $t_{y}$ denote the longitudinal and lateral translations between the two maps, and ${\left[x,y,1\right]}^{T}$ is the homogeneous coordinate representation of any point (x,y) in map m.

One approach can be devised for solving the map-merging problem based on map overlapping. In this approach, the map-merging problem is equivalent to finding an optimal transformation to maximize the extent of coincidence between two maps. Therefore, Carpin et al. [21,33] defined the overlapping between two maps m1 and m2 as follows:

(8) $$\omega (m1,m2)=\sum_{i=0}^{N-1}\sum_{j=0}^{M-1}Eq(m1[i,j],m2[i,j])$$

where Eq(a,b)=1 when a=b, otherwise, Eq(a,b)=0. The overlapping function ω() can be regarded as the objective function in the optimization problem. To obtain the optimal transformation $T_{\left(x,y,\theta \right)}$, one only needs to maximize the overlapping function $\omega (m1,T_{\left(x,y,\theta \right)}(m2))$.

Existing solving algorithms for the above optimization problem include simulated annealing [34] and multi-point hill climbing [35]. Besides, Carpin et al. [33] proposed a more general motion planning algorithm-the adaptive random walk (ARW) algorithm-to solve this optimization problem. However, this algorithm is essentially based on iterative exhaustive search, whose computational load is fairly large. Considering this shortcoming, Birk and Carpin [21] used an heuristic function to guide the search process and provide feedback for the continuing direction of the search algorithm, which effectively speeds up the search process. Then, an error detection mechanism is proposed in this work to effectively avoid a false map merging and enhance algorithm robustness. However, the fusion results of this algorithm are compromised if the extent of overlapping between the two maps is very low.

As mentioned above, the computation load of the ARW algorithm proposed in [33] is fairly large. Aiming to overcome this shortcoming, Ma et al. [36] proposed a nonlinear adaptive genetic algorithm to merge occupancy grid maps. The genetic algorithm imitates the natural evolutionary procedure and has the characteristics of random global search and parallelism. It is very suitable for finding the target solution in a large number of possibilities, and it achieves the optimal match between two maps more effectively than the random walk algorithm.

#### 2.2.2. Objective Function Based on Occupancy Likelihood

Li and Nashashibi [37,38] employed occupancy likelihood in the objective function design, which is similar to the idea introduced in [21,33]. In their approach, the level of consistency between two occupancy grid maps $m_{A}$ and $m_{B}$ is measured by the following objective function Fc:

(9) $$Fc(m_{A},p_{\mathrm{BA}}⊕m_{B})=\sum_{i=1}^{n}\{m_{A}(p_{\mathrm{BA}}⊕o_{B(i)})|p_{\mathrm{BA}}⊕o_{B(i)}\mathrm{is}\;\mathrm{occupied}\;\mathrm{in}\;m_{A}\}$$

where $o_{B(i)}$ represents the set of occupied grids in $m_{B}$, and $p_{\mathrm{BA}}$ stands for the transformation matrix. Note that the grid cell positions in $o_{B(i)}$ and $m_{B}$ are both denoted as points in a two-dimensional space, and the elements in $p_{\mathrm{BA}}$ can be expressed in the form of a 3-by-1 vector. Following this convention, the compounding operation ⊕ in Equation (9) is given by:

(10) $$\left[\begin{array}{c} x_{1}\\ y_{1}\\ \theta_{1} \end{array}\right]⊕\left[\begin{array}{c} x_{2}\\ y_{2} \end{array}\right]=\left[\begin{array}{c} x_{2}\cos\theta_{1}-y_{2}\sin\theta_{1}+x_{1}\\ x_{2}\sin\theta_{1}+y_{2}\cos\theta_{1}+y_{1} \end{array}\right]$$

Then, the optimal transformation matrix $p_{\mathrm{BA}}$ is solved to maximize the consistency between $m_{A}$ and $p_{\mathrm{BA}}⊕m_{B}$, namely:

(11) $${\hat{p}}_{\mathrm{BA}}=\underset{p_{\mathrm{BA}}}{\arg\max\;}Fc(m_{A},p_{\mathrm{BA}}⊕m_{B})$$

Lastly, the genetic algorithm is applied to solve the objective function, and the initial value of $p_{\mathrm{BA}}$ is obtained through GPS positioning. Experiment results show that this method finds out the optimal solution within a very short period of time (no more than one second).

Based on the above work, Li et al. [10] further proposed an indirect vehicle-to-vehicle (V2V) relative pose estimation strategy. This approach is able to estimate the relative poses between vehicles without knowing their initial positions, and it can be used as a general solution for multi-vehicle data association.

#### 2.2.3. Objective Function Based on Image Registration

In the above works, an occupancy grid map is represented by a matrix that stores the probability of each cell being occupied. If each value in this matrix is considered as a pixel in the image, then an occupancy grid map can be indeed represented by an image. Therefore, the problem of an occupancy grid map merging can be transformed to an image registration problem in computer vision. The process of occupancy grid map merging based on image registration is explained as follows [39].

It is assumed that there exists some overlapped area between the two maps to be merged. The edge points extracted from the two maps are represented by two sets $P={\left\{p_{i}\right\}}_{i=1}^{Np}$ and $Q={\left\{q_{j}\right\}}_{j=1}^{Nq}$, where $N_{p}$ and $N_{q}$ are the number of elements in set P and set Q, respectively. Now, the process of occupancy grid map merging is equivalent to computing the rigid transformation T={R,t}, so that the transformed edge points set T(P) well matches the edge points set Q. The above merging process can be expressed as a mathematical minimization problem, given by [39,40]:

(12) $$\begin{array}{l} \min_{\underset{c(i)\in \{1,2,\cdots ,Nq\}}{R,t,\xi ,P_{\xi }}}\frac{1}{|P_{\xi }|{\left(\xi \right)}^{1+\lambda }}\sum_{p_{i}\in P_{\xi }}∥Rp_{i}+t-q_{c(i)}{∥}_{2}^{2}\\ s.t.\;R^{T}R=I_{2\times 2},\;\det(R)=1\\ \;\xi \in [\xi_{\min},1],\;P_{\xi }\subseteq P,\;|P_{\xi }|=\xi |P| \end{array}$$

where λ is a control parameter, |⋅| denotes the cardinality of the set (i.e., the number of elements in the set), $\xi_{\min}$ represents the minimum allowed overlap percentage, c(i) represents the index of elements in set Q, and $P_{\xi }$ denotes the subset of P, which corresponds to the common part (intersection) of sets P and Q.

To solve the above equation, the iterative closest point (ICP) algorithm [41] and the trimmed ICP (TrICP) algorithm [42] have been proposed. Aiming to enhance the efficiency of the above algorithms, Zhu et al. [40] proposed an improved TrICP algorithm, which effectively prevents the optimization function from obtaining a local optimal solution and in turn ensures fast acquisition of the global optimal rigid transformation T.

### 2.3. Feature-Based Method

Considering a multi-robot SLAM scenario, the individual robots construct their own occupancy grid maps in the SLAM process. When fusing the maps generated by these robots, the points of interest (i.e., features) of the constructed maps are first extracted, and then these extracted features are used to merge the occupancy grid maps [43]. The extracted features are usually considered static and do not change with time, ensuring that the extracted features can appear repeatedly in different local maps [26]. Common types of features include point features (e.g., scale-invariant feature transform (SIFT) features [24,44], speeded-up robust features (SURF) [45], and Harris [46]), line features (e.g., line segments [47], arcs [47]), and geometric features (e.g., rectangles [48]).

Wang et al. [44] regarded occupancy grid maps as images. They extracted SIFT features from images and merged local maps using the ICP scan matching algorithm [41]. The reliability of the proposed algorithm is verified using the simulation platform, Robot Technology Middleware (RTM). Sun et al. [49] proposed an occupancy grid map fusion algorithm based on maximum common subgraph (MCS) [49]. In this approach, the Harris corner points are first extracted in the local maps, and then the maximum common subgraph is obtained through iterative algorithm. Lastly, based on the relationship between the corner points, a transformation matrix is calculated to merge the maps. One shortcoming of this method is that it becomes inefficient when too many corners are extracted. The high-frequency noise existing in the occupancy grid maps constructed by the laser scanners can lead to false features in the feature extraction results, which seriously jeopardizes the accuracy of map merging. To overcome this shortcoming, Blanco et al. [26] proposed a pre-processing method for occupancy grid map images by smoothing the images using a Gaussian filter and a median filter successively before feature extraction. This method has been applied to different types of features such as SIFT, SURF, Harris, and KLT (Kanade-Lucas-Tomasi). The results show that this method greatly improves the detection quality of SIFT and SURT features, while the effects on Harris and KLT features are not obvious.

Although numerous solutions have been proposed in the literature for merging occupancy grid maps, it is still highly challenging to deal with occupancy grid maps with different resolutions. For merging maps with the same resolution, the transformation involved is a rigid transformation. However, when dealing with maps with different resolutions, the rigid transformation T={R,t} changes to a scaling transformation T=(s,R,t) [39], where s is a scale factor. The existing methods (e.g., [24,39]) for merging maps with different resolutions mainly rely on the extraction of map features. Liang et al. [39] present a novel scaling registration approach for merging occupancy grid maps with different resolutions, however this method can only merge two maps at a time. To solve this problem, Jiang et al. [24] proposed a map augmentation and feature fusion (MAFF) strategy to fuse several maps with different resolutions. Since the local maps with different resolutions must be merged into the global map, the features extracted from the local maps should be invariant to scale, rotation, and translation changes. As a result, the SIFT features, which are geometrically invariant under different scale transformations, are adopted in the above two methods. However, one drawback of using SIFT features is that the local maps cannot be merged into the global map, if the SIFT features are not extracted from the local maps. To tackle this issue, Topal et al. [50] calculated the transformation between maps by extracting another type of scale-invariant features called “key points”. By this means, the occupancy grid maps can be better combined in an unstructured environment with less overlap.

In addition to the traditional point features mentioned above, other types of features are also discussed in the latest research. Alnounou et al. [47] used the Hough transform to extract line segments and circles from occupancy grid maps and employed them as features. By this means, real-time map merging can be realized for the outdoor unstructured environment, and the map-merging results are validated using the validation criteria proposed in [21]. Since occupancy grid maps can be represented by a binary matrix, Park et al. [48] proposed a map-merging method by extracting the maximal empty rectangular (MER) features from the local maps. The information of robot position is not necessary in this approach; however, it requires at least three empty rectangles in the local map. This method is also called the “R-map” algorithm. For more detailed information, interested readers are referred to [51].

None of the above feature-based methods has taken into account an extreme case-mismatching of features. This mismatching happens when two similar features in different maps are mistaken for the same feature [46]. Mismatching of features can often lead to serious map-merging faults, which commonly occur in environments with high repeatability such as symmetrically structured environments. To tackle this issue, Durdu and Korkmaz [46] proposed a semantic approach to fuse environments with symmetric parts. This approach effectively avoids erroneous feature matching in symmetric environments by evaluating the effectiveness of features. Besides, it is also able to merge maps in environments where SURF, features from accelerated segment test (FAST) [52], or other types of features are mismatched.

### 2.4. Hough-Transform-Based Method

Both the probability-and optimization-based map-merging methods are essentially algorithms relying on iterations. Due to the nature of these algorithms, the computation load inevitably increases as the environment grows. Considering this drawback, Censi et al. [53] first applied the Hough transform [54] to solve the scan matching problem. Inspired by this work, Carpin [55] proposed a non-iterative algorithm, in which the Hough transform is employed to extract linear features and a series of possible transformations are generated. Then, an acceptance index function is designed to weight the potential transformations, thereby leading to an optimal transformation. This algorithm is suitable for real-time map merging because of its fast speed and high robustness. However, it requires a large overlap between the two local maps to be merged. Moreover, affected by the Hough transform discretization error, the accuracy of this method needs further enhancement. In view of these limitations, Saeedi et al. [56,57] improved Carpin’s algorithm and employed a similarity index [21] to verify the performance of map merging. In the case of low map overlap, this algorithm ensures satisfactory merging performance for occupancy grid maps.

## 3. Feature-Based Map Merging

**Figure 4:**
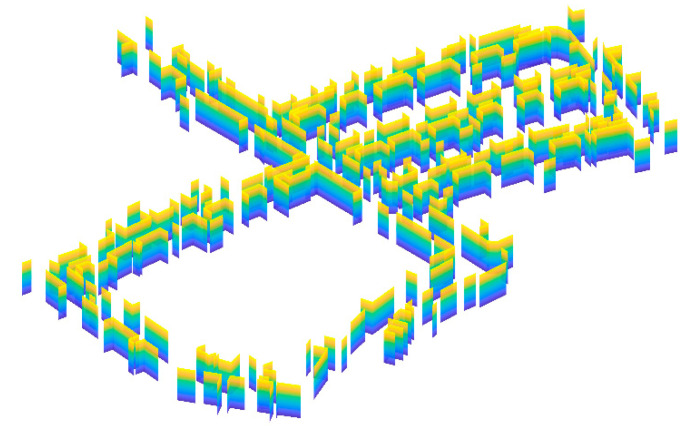
A plane-feature-based map example.

Since occupancy grid maps do not involve feature extraction and provide a direct representation of the environment, they have attracted a lot of attention from the robotics community [21,39,58]. However, occupancy grid maps are indeed a type of redundant map representation, which inevitably requires a large amount of memory and computation power. For this reason, more and more studies have been focused on extracting features from the environment and building feature-based maps of the environment. Commonly used features include point features [59,60,61], line features [62,63,64,65,66], and plane features [67,68,69,70]. A plane-feature-based map is shown in Figure 4. In structured environments, numerous objects (such as walls and buildings) can be represented by lines and planes. As a result, line features and plane features present obvious advantages for providing a compact map representation and avoiding data redundancy.

In the existing literature, three types of map features (i.e., points, lines, and planes) are mainly used to construct feature-based maps. On this basis, the following subsections will discuss the existing map-merging methods for the three types of features.

### 3.1. Point-Feature-Based Map Merging

Compared with other features (e.g., line features and plane features), point features are the most straightforward and commonly used feature type. Point features can be easily employed to represent unstructured environments, but maps using point features are generally more sensitive to noise. Common types of point features include SIFT [71], SURF [45], and oriented FAST and rotated binary robust independent elementary features (BRIEF) (ORB) [72].

It should be noted that in point feature maps, the environments are not represented by the extracted feature points only. Indeed, the environments are usually represented directly by dense point clouds. These point clouds can be obtained by Lidar sensors or depth cameras. The extracted feature points are normally used in the following two cases: (1) inter-frame feature matching in the local map construction process of a single robot and (2) calculation of rigid transformation between local maps produced by different robots.

For point-feature-based maps, a key step in the map-merging process is to extract stable point features. This is because unstable point features can lead to unreliable feature matching results, which in turn jeopardizes the map-merging performance. Konolige et al. [31] attempted to match the manually extracted features (such as doors, intersections, and corners) in two local maps for map merging, in the event that stable point feature extraction cannot be guaranteed. Sun et al. [61] employed the open source algorithm ORB_SLAM2 [60] to extract FAST key points [73] and binary robust independent elementary features (BRIEF) descriptors [74] for building sparse point cloud maps. The point features extracted from the local maps are compared with those in the off-line dictionary based on the bag of word (BoW) model [75]. By this means, the features in two local maps can be matched more quickly, which in turn accelerates the acquisition of the global map.

### 3.2. Line-Feature-Based Map Merging

Compared with point features, the process of line feature extraction is less susceptible to noise [76]. Line features have been used in indoor environments, as they are ubiquitous in indoor scenarios, such as junctions of walls and edges of tables. So far, no relevant studies have been found in the literature that employ only line features to represent the outdoor environments. Limited by the sensor field of view, in outdoor environments, it is highly likely for the sensor to observe only a portion of the entire line feature in a single frame. The complete observation of a large line feature often requires more than one frame. This phenomenon brings about new challenges for map merging: inter-frame feature matching. Six typical algorithms for extracting straight lines from 2D Lidar data are summarized in [77], which are split-and-merge algorithm, incremental algorithm, Hough transform algorithm, line regression algorithm, random sample consensus (RANSAC) algorithm, and expectation–maximization (EM) algorithm. For brevity, the details of these algorithms are not repeated in this paper, and interested readers are referred to [77].

In the existing literature, it is commonly assumed that the initial relative poses of robots are known [78], or that the relative poses can be measured by onboard sensors when the robots encounter each other [11]. Amigoni et al. [79] proposed a method for merging two local maps composed of line segments. This method is composed of three major steps: (1) find possible transformations, (2) evaluate all transformations, and (3) determine the best transformation and fuse the line segments. In a follow-up study [66], Amigoni et al. extended the application of the above approach to arbitrary numbers of maps without the information of position relationships between local maps. Three different map fusion sequences are proposed in this work, which are the sequential method, the tree method, and the pivot method.

To merge feature-based maps, the conventional approach [66,79] is to calculate the relative transformation between local maps through feature matching. Inspired by the curve matching framework introduced in [80], Baudouin et al. [81] proposed a novel way for solving the map-merging problem. By calculating and matching the curvature of each point on the robots’ motion trajectories, the rigid transformation between robot trajectories can be obtained for map-merging purposes. In this approach, robot trajectory matching is employed instead of feature matching, which avoids the adverse influence of unstable features on the calculation of transformation between maps.

For line-segment-based maps, the key problem lies in the fusion of redundant line segments for both single and multi-robot systems. The main difficulty of merging redundant line segments is to determine whether multiple line segments from different sources belong to the same structure [82]. This difficulty commonly arises when dealing with the following situations: different line segments coming from different frames in a single-robot system, or different line segments coming from different robots in a multi-robot system. To overcome this difficulty, Amigoni and Li [64] reviewed the commonly used methods for merging line segments in the existing literature, such as [81,82,83,84,85,86,87], and then evaluated the performances of these methods using open datasets [88]. In short, these methods can be roughly divided into two categories. The first category (e.g., [83,84,87]) determines whether different line segments represent the same object according to the geometric relationships between the line segments (such as the maximum distance between endpoints and the included angle difference of lines relative to the X-axis). The principle of these methods is straightforward and intuitive, but the reduction rate of line segments in the final fused map is low. The second category (e.g., [85,86]) clusters line segments based on the mean shift algorithm [89] and then merges these line segments. Due to the clustering process, the final fused map is more compact but at the cost of longer computation time.

### 3.3. Plane-Feature-Based Map Merging

The size of explored environment and efficiency of map construction can be significantly enhanced when a multi-robot SLAM system is employed. However, if raw sensor data are utilized to represent maps for multi-robot SLAM, large capacity storage and high-speed onboard computation devices are necessary, as the amount of data involved in raw sensor measurements is often enormous. In view of this shortcoming, more sophisticated features (such as plane features [90]) are extracted from the environment for map representation. Although more sophisticated features normally lead to less amount of data, the efforts spent on feature extraction are also higher.

Before the advent of depth cameras, accurate plane features were mainly extracted from Lidar point cloud data. For instance, the plane features can be obtained by fitting a plane model in a large amount of 3D points. As a result, these features are less susceptible to noise and in turn present better robustness [91]. The statistical tools used for plane extraction mainly include RANSAC [92] and the modified selective statistical estimator (MSSE) [93]. Apart from 3D Lidar sensors, low-cost 2D laser range finders can also be employed to collect point clouds, if they are installed aslant or rotated during operation [68,94]. In recent years, along with the increasing application of depth cameras, ordered 3D point clouds obtained by depth cameras have been used for plane extraction [95]. This approach is faster than extracting planes from unordered point clouds collected by Lidar sensors, and real-time plane segmentation can be achieved at a frame rate of 30 Hz [96].

Similar to the line features introduced above, if a large planar structure is encountered by a robot, it is highly likely that only part of the plane is observed in each frame due to the limitation of sensor FOV. Thus, features that originate from the same planar structure may repeatedly appear in several consecutive frames, and these redundant features must be fused to reflect a single planar structure in the environment. A similar problem appears in the map-merging process of multi-robot SLAM. Namely, one needs to determine whether the plane features in different local maps generated by different robots originate from the same structure in the environment. Then, the features representing the same structure are merged to obtain an updated global map. In order to merge planes segmented in different frames or merge the local maps generated by different robots, it is highly necessary to transform the planes into the same coordinate system to simplify computation. Gostar et al. [97] proposed a plane transition model to convert the plane parameters in the vehicle coordinate system to the global coordinate system. This method can be employed to transform the planes obtained in different frames into the same coordinate system for map-merging purposes.

As mentioned above, the key problem existing in plane feature-based map merging is to determine whether the plane features obtained by different robots originate from the same structure in the environment. Lopez-Sanchez et al. [67] employed a set of planes to represent indoor structured environments. These environments are assumed to be strictly orthogonal, namely, the angle between any two connected obstacles is 90°. This approach is able to determine whether the plane features by different robots represent the same planar structure, through calculating the distances between the plane features. Due to the strict assumption on orthogonality, the application of this method is limited. Lenac et al. [98] projected 3D point clouds onto the 2D image planes and then calculated the plane parameters from clustered point clouds by means of least square fitting. Different from directly converting the extracted planes to a uniform coordinate system, in this work, the plane features representing the same structure are merged based on the plane uncertainty model. By this means, the number of planes is greatly reduced, and the SLAM process is accelerated.

In earlier works, the extracted plane features usually do not contain any boundary information, and as a result, the size of planes is indeed infinite. Not only does this plane representation a hamper plane merging but also neglects important dimensional information about the environment. Zureiki and Devy [99] acquired the plane boundaries by extracting straight line features from an image. Trevor et al. [100] obtained plane parameters from 3D point clouds using the RANSAC approach. Then, a convex hull is calculated from the inner points in a plane to define the plane boundaries. In this method, the plane parameters and the convex hull should be used together to represent a plane feature. When a plane is re-visited, the convex hull is then updated with newly observed points belonging to the plane, which in turn leads to a complete plane structure.

## 4. Topological Map Merging

**Figure 5:**
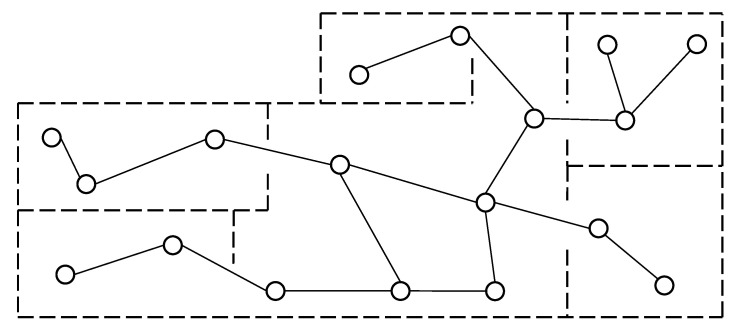
A topological map example.

Compared with occupancy grid maps and feature-based maps, topological maps are less intuitive in terms of environment representation. As a result, the number of relevant works in the existing literature is a lot fewer compared to other map types. In topological maps, nodes are employed to represent places/features in the environment, and edges connecting these nodes are used to represent paths between these places/features. The nodes in the topological maps are normally unique, i.e., each node has a unique label, and the edges connecting these nodes contain connectivity and distance information between the places/features. The above attributes give topological maps unique advantages in such applications as localization [101,102,103], navigation [104], and path planning [105,106]. For example, given the starting and ending places, popular path planning algorithms such as Dijkstra [107] and Floyd [108] can be readily employed to calculate the shortest path, while other types of maps have no such advantage. Common types of topological maps include: Voronoi graph (VG) [109], generalized Voronoi graph (GVG) [102], annotated generalized Voronoi graph (AGVG) [110], and extended Voronoi graph (EVG) [111]. A typical topological map is shown in Figure 5.

In the existing literature, the topology information contained in topological maps is mainly utilized for map-merging purposes, when the environment is only represented by topological maps (i.e., no other types of maps involved). Huang and Beevers [109] proposed an algorithm, which utilizes both map structure and map geometry to identify possible matches and directly merges topological maps based on overlapping areas. Although this algorithm can effectively reduce computation load, it cannot be used for topological map merging in dynamic environments where moving objects exist (i.e., the positions of objects change with time). Ferreira et al. [103] proposed a method to construct topological maps for large-scale environments by directly merging multiple topological paths without constructing occupancy grid maps or feature-based maps first. This method is more efficient for large-scale environments, since only topological maps are used, and no other map types are involved. However, this approach regards each frame as a node, which inevitably results in more redundant information.

Since the details of environments are lacking in topological maps, hybrid maps have been proposed in the literature to combine other types of maps and topological maps [112]. Bernuy and Ruiz-del-Solar [113] proposed a SLAM solution for large-scale outdoor scenes, based on topological semantic maps. This method employs particle filtering to locate one robot in the map of another, which in turn provides reliable transformation for map fusion. Chang et al. [112] proposed a map fusion algorithm based on topology/metric maps. In this work, the robot system is completely distributed (i.e., no central robot), and each robot plays the same role in the system. In the SLAM process, each robot constructs a local map of the environment along its own path. When adjacent robots are within the communication range of each other, they exchange their map information, and the local maps are then merged into a global map.

## 5. Summary

Along with the rapid advancement of autonomous driving and robotic technologies, multi-robot systems, as a feasible solution to large-scale outdoor scenarios, have attracted increasing attention from both academia and industry. In order for a cooperative multi-robot system to work effectively, the local maps generated by individual robots must be properly fused/merged. Therefore, the map-merging methods play a critical role in the operation of a cooperative multi-robot system.

This paper reviews the existing map-merging methods for three major types of maps used in SLAM applications, i.e., occupancy grid maps, feature-based maps, and topological maps. The occupancy grid maps, as the earliest map type used, have been studied extensively in the literature. The merging methods for occupancy grid maps mainly include the probability method, optimization method, feature-based method, and Hough-transform-based method. Compared to occupancy grid maps, feature-based maps can represent the environmental more efficiently. Although the number of existing studies on feature-based map merging is quite low so far, the use of feature-based maps for environment representation is a promising trend in the future. The existing merging methods for feature-based maps (including point, line, and plane feature maps) are introduced in this paper. Compared to the first two types of maps, topological maps are more abstract and focus mainly on the topological information of the environment. These maps present unique advantages in path planning and navigation, which make themselves an important map type in the literature. The existing merging methods for topological maps are also reviewed in this paper.

At present, occupancy grid maps are the most mature map type, and numerous map-merging methods have been proposed in the literature. In comparison, feature-based maps and topological maps are relatively newer, and the number of related works are also a lot fewer. However, in recent years more attention has been paid to these new map types due to their effectiveness and advantages. In addition, the above maps can also be used jointly to form a hybrid map for environment representation, such as the combination of feature-based map and topological map. It is predicted that we will see more applications of feature-based maps, topological maps, and hybrid maps in the near future.

## Abbreviations

The following abbreviations are used in this manuscript:

SLAM	simultaneous localization and mapping
ARW	adaptive random walk
ICP	iterative closest point
TrICP	trimmed ICP
SIFT	scale-invariant feature transform
SURF	speeded-up robust features
KLT	Kanade-Lucas-Tomasi
MCS	maximum common subgraph
MAFF	map augmentation and feature fusion
MER	maximal empty rectangular
FAST	features from accelerated segment test
ORB	oriented FAST and rotated BRIEF
BoW	bag of word
RANSAC	random sample consensus
EM	expectation–maximization
MSSE	modified selective statistical estimator
VG	Voronoi graph
GVG	generalized Voronoi graph
AGVG	annotated generalized Voronoi graph
EVG	extended Voronoi graph
